# Taro Roots: An Underexploited Root Crop

**DOI:** 10.3390/nu15153337

**Published:** 2023-07-27

**Authors:** Md. Jannatul Ferdaus, Ezzine Chukwu-Munsen, Aline Foguel, Roberta Claro da Silva

**Affiliations:** 1 Family and Consumer Sciences, College of Agriculture and Environmental Sciences, North Carolina A&T State University, Greensboro, NC 27411, USA; mferdaus@aggies.ncat.edu (M.J.F.); ecchukwu@aggies.ncat.edu (E.C.-M.); 2 Department of Biochemical-Pharmaceutical Technology, Faculty of Pharmaceutical Sciences, University of Sao Paulo, Sao Paulo 05508-000, SP, Brazil; alinefoguel@usp.br

**Keywords:** taro roots, dietary fiber, product development, utilization, nutritious, antinutritional, underutilized, taro-based products

## Abstract

Taro (*Colocasia esculenta*) is a root crop that remains largely underutilized and undervalued despite its abundance and affordability. In comparison to other root vegetables, such as potatoes, yams, carrots, and cassava, taro stands out as a plentiful and low-cost option. As global hunger increases, particularly in Africa, it becomes essential to address food insecurity by maximizing the potential of existing food resources, including taro, and developing improved food products derived from it. Taro possesses a wealth of carbohydrates, dietary fiber, vitamins, and minerals, thereby making it a valuable nutritional source. Additionally, while not a significant protein source, taro exhibits higher protein content than many other root crops. Consequently, utilizing taro to create food products, such as plant-based milk alternatives, frozen desserts, and yogurt substitutes, could play a crucial role in raising awareness and increasing taro production. Unfortunately, taro has been stigmatized in various cultures, which has led to its neglect as a food crop. Therefore, this review aims to highlight the substantial potential of taro as an economical source of dietary energy by exploring the rich fiber, potassium, vitamin C, protein, and other micronutrient content of taro, and providing a foundation for the formulation of novel food products. Furthermore, this paper assesses the nutritional benefits of taro, its current utilization, and its antinutritional properties. It emphasizes the need for further research to explore the various applications of taro and improve on-farm processing conditions for industrial purposes.

## 1. Introduction

Roots and tubers are recognized as substantial contributors to global carbohydrate consumption and are second only to cereals. These crops are cultivated extensively in developing nations, such that millions of farmers rely on them for food security and economic prosperity [1]. These crops are vital to income generation, sustainable development, and household food security, particularly in low-income countries. Moreover, they find versatile applications in animal feed, starch extraction, and the production of fermented foods and beverages [2]. The most world-consumed tropical roots and tubers are taro (*Colocasia esculenta*), yam (*Dioscorea* spp.), potato (*Solanum tuberosum* L.), sweet potato (*Ipomoea batatas*), cassava (*Manihot esculenta*), and elephant foot yams (*Amorphophallus paeoniifolius*). Taro holds great promise as a food and has the potential to combat malnutrition, given its underutilization thus far [3]. The taro crop has enormous potential as an economical source of dietary energy, starch, fiber, potassium, vitamin C, protein, and other micronutrients [4]. 

The taro plant belongs to the Araceae, which are aroids grown principally for their edible corms, petioles, and leaves. The Araceae family has 110 genera and more than 2500 species, which include Colocasia, Xanthosoma, Amorphallus, Alocasia, and Cytosperma [2]. The polymorphic species Colocasia esculenta, often known as eddo or dasheen, is cultivated for its tasty corm and is exported internationally. *Colocasia esculenta* (L.) Schott var. esculenta and *Colocasia esculenta* (L.) Schott var. antiquorum (School) are the two widely available varieties [5].

There are potentially thousands of known taro cultivars that fall into two categories: (1) the “eddoe” type (*Colocasia esculenta* var. antiquorum syn. *Colocasia esculenta* var. globulifera) and (2) the “dasheen” type (*Colocasia esculenta* var. esculenta) [6]. These two main varieties present different corm types; the dasheen variety is long central with few side-corms (cormels), whereas the eddoes variety shows a well-developed cormel.

**Figure 1:**
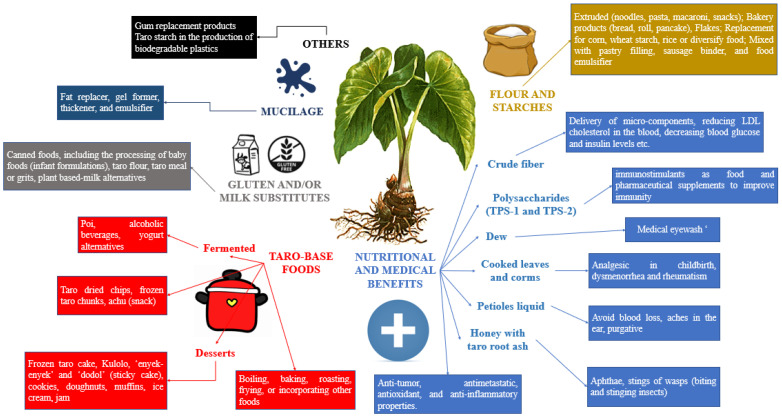
Clinical benefits and usages of taro in different food products.

Taro plants exhibit adaptability to a wide range of soil conditions, spanning from well-drained dry soils to waterlogged soils in high-rainfall areas. Optimal taro growth occurs in warm and humid environments, which is characterized by mean temperatures that range from 25 to 30 °C and well-distributed rainfall [7]. However, supplemental irrigation is occasionally necessary to ensure a successful yield. Considering the sustainability and high nutritional value of taro roots, it is worth exploring their potential to thrive in warmer regions of the USA. In addition, taro stands out as a root crop with zero waste since every part of the plant, including the leaves, peel, and root, can be utilized [8]. Nevertheless, it is unfortunate that only a limited number of studies have investigated the industrial utilization of taro as a source of ingredients for processed food products. The divisible usages of taro and its health benefits are demonstrated in Figure 1.

## 2. Origin and Production in the World

Taro (*Colocasia esculenta* (L.) Schott) is a tropical root crop with a rich history, which belongs to the Araceae family and encompasses over 10,000 landraces. It is cultivated worldwide in tropical and subtropical regions, including Africa, China, New Guinea, various Pacific islands, all Caribbean islands, parts of Central and South America, as well as specific regions in the United States. Recognized as one of the oldest known crops, taro is believed to have been domesticated more than 10,000 years ago [9,10]. Archaeological evidence from the Solomon Islands suggests its utilization dates back nearly 28,000 years [11]. However, establishing a single center of origin for taro has proven challenging. Some theories propose South Central Asia, specifically India or Malaysia as the possible origin [12]. Matthews (1990) hypothesized an origin between Myanmar and Bangladesh in the Indo–Malayan region [13]. Nevertheless, with a comprehensive genetic analysis of cultivars or wild taro materials from these distributed regions, the precise origin of the crop remains to be determined [10].

**Figure 2:**
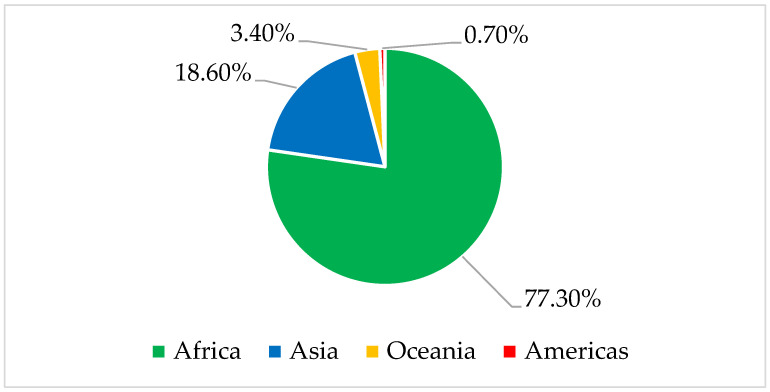
Production share of taro by region in 2021. Source: FAOSTAT 2023 [14].

Taro is a widely cultivated root crop in 50 countries worldwide. Nigeria is the largest producer of taro, followed by Cameroon, China, and Ghana, and collectively, they account for over half of the global taro production (Figure 1). In 2021, global taro production reached 12,396,248.5 tons, with Africa contributing 77.30% of that output. Asia contributed 18.60%, while Oceania and the Americas contributed 3.40% and 0.70%, respectively (Figure 2). 

Table 1 provides the annual taro production for various regions worldwide. In 2000, Africa was the top producer with 8,233,653.65 tons, followed by Asia with 1,930,699.73 tons. As of 2021, Africa continued to lead, producing 9,525,695.56 tons, despite a slight drop in 2010 (to 7,754,061.42 tons). The Americas had the lowest taro production from 2000 to 2021, although they experienced a substantial increase in 2015, when they produced 133,346.23 tons.

**Figure 3:**
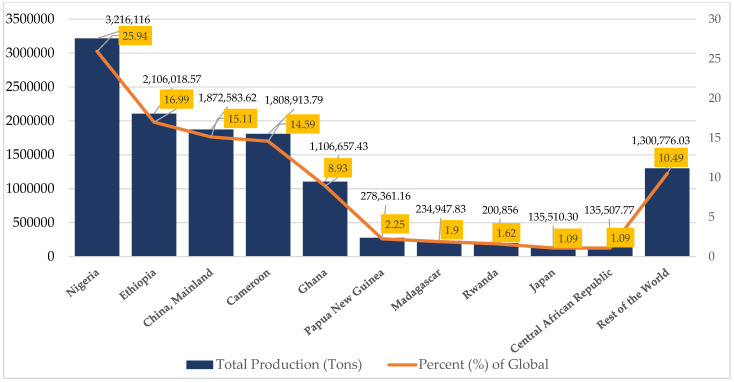
Top 10 taro producers in 2021. Source: FAOSTAT 2023 [14].

Figure 3 clearly indicates that the major share of global taro production in 2021 was dominated by 10 countries and accounted for nearly 90%. Nigeria produced the most with 25.94%, while China and Cameroon made comparable contributions of 15.11% and 14.59%, respectively. Conversely, Japan and the Central Africa Republic displayed a significantly lower contribution, each contributing a mere 1.09% to the overall global taro production.

Taro cultivation is typically carried out on a small scale by impoverished rural African women with limited resources. Despite being the third most important root and tuber crop after cassava and yams, taro must be utilized for export and industrial purposes, considering its nutritional benefits for health in Africa. On a global scale, the United States is the largest importer of taro, importing 35.7% of the total production in 2021 [15]. However, North America and the Asia–Pacific region are the primary suppliers of taro roots worldwide. Taro is also commercially grown in Hawaii and various parts of the Pacific Basin, although cultivation is usually limited to small plots near households. Hawaii is the largest taro producer in the US, with an estimated 4.8 million pounds of taro produced in 2021 [16]. In ancient times, Hawaiian planters cultivated around 300 taro species, often distinguished by the colors of distinct leaf portions [17]. However, taro is also grown on small-scale farms in California.

Taro can be broadly categorized into two types: upland taro, which is commonly used as a potato substitute, and wetland taro, which is primarily utilized for making poi, a fermented taro dish. While the long corms, narrow stems, and leaves of the taro plant are edible, they require thorough cooking before consumption to ensure that they are safe to eat and to enhance their taste and texture.

## 3. Nutritional Composition of Taro

Taro is a valuable source of starch, which is obtained from its underground stems or corms. Additionally, its leaves and flowers act as nutritious vegetables [18]. The nutritional composition of taro corms varies significantly depending on factors including the variety of taro, its country of origin, soil quality, growing conditions, and environmental interactions. These factors can influence important nutritional components, such as vitamins, minerals, fiber content, and overall nutrient density [19]. For instance, Huang et al. [20] found that the Bun-long taro variety exhibited significantly higher protein, ash, and macro-mineral contents than the Hawaiian varieties. Furthermore, the authors observed that both taro varieties had higher dietary fiber contents than the USDA data for taro. Plant age is another crucial factor since different growth stages significantly impact the nutritional composition of taro. It has been observed that immature roots tend to have lower water content and higher starch content compared to mature roots. Additionally, other nutritional components, such as fiber, minerals, and vitamins, may also vary depending on the plant’s age and development. Taro provides approximately 135 kcal per 100 g, while it is typically low in protein, with a content of 11% in dry weight, and low in fat (0.3–0.6%). However, it is a good source of carbohydrates, vitamins, and minerals [21,22]. The proximate composition of the taro roots and leaves of different studies is represented in Table 2.

### 3.1. Moisture Content

The taro moisture content is significantly important due to its implications on texture, taste, and susceptibility to spoilage. Higher moisture levels contribute to a softer and more delicate texture, which can be desirable in specific culinary applications. However, these conditions also increase the risk of microbial growth and enzymatic activities, leading to spoilage and a decline in quality over time [28]. Taro and other edible aroids are challenging to store and transport due to their bulk and vulnerability to physical damage, which allows microbes to invade and cause decay in the interior starchy tissues [4].

The moisture content of the roots and tubers directly affects their overall yield and market value. Excessive moisture loss during storage or transportation results in weight reduction and physical damage, which results in economic losses for producers and suppliers. Hence, optimizing storage conditions and implementing appropriate handling practices are crucial to maintaining the taro’s moisture content and maximizing its market value [28]. 

To reduce the challenge of the short shelf life, diverse post-harvest techniques and technologies have been explored. These encompass modified atmosphere packaging, temperature and humidity control, and the use of natural and synthetic preservatives. These approaches aim to extend the taro shelf life by minimizing moisture loss, inhibiting microbial growth, and reducing enzymatic reactions [4]. Refrigeration storage can be employed to preserve the taro and extend its shelf life. For instance, research suggests that taro corms can be stored successfully for 5–6 weeks at 15 °C and 85% relative humidity, or in a well-ventilated chamber at 7–12 °C for at least 8 weeks without significant quality degradation [29]. Similarly, taro roots can be preserved for approximately 8 weeks at temperatures of 0 to 2 °C and 2 to 4 weeks at 20 °C. However, roots stored at room temperature should be consumed within two days [30]. 

Packaging also plays a critical role in preventing the deterioration of taro corms. Common packaging practices in the U.S. involve the use of 50-pound containers with adequate ventilation. Smaller roots may be sold in 10-pound boxes to increase the sale and utilization. Perforated polymeric films and polyethylene have been reported as effective in regulating the moisture content of taro roots, along with the combination of hot water dipping and storage at 4 °C for 12 days to effectively inhibit browning and extend the shelf life of peeled taros [31,32].

Through the implementation of effective post-harvest strategies and meticulous handling, the challenges arising from the taro’s high moisture content can be mitigated. This versatile crop can maintain its freshness and value by ensuring proper practices throughout the supply chain, contributing to its successful marketability and overall viability. These advancements in post-harvest technologies reduce losses and improve shelf life, storage, and processing, which ultimately contributes to food and nutritional security [31].

### 3.2. Carbohydrates 

The taro’s high carbohydrate content (86.11 ± 0.06%) provides an excellent energy source, with a starch content of 70–80% in the dry roots [33]. Therefore, starch in taro has a high glycemic index. Nevertheless, extended storage and precooking treatments can generate highly resistant starch in taro flour. Resistant starch has been linked to slower digestion in the lower regions of the human intestinal tract, thereby contributing to the slower release and absorption of glucose, which helps to reduce the chance of diabetes, obesity, and other linked diseases [21]. Simsek and Nehir El [34] conducted a study where they successfully produced resistant starch from natural taro, demonstrating its potential to reduce the glycemic index and bile acid-binding capacity compared to natural taro starch. Additionally, Emmanuel-Ikpeme [35] suggested that taro starch was associated with other health benefits, such as in patients with an ulcer, pancreatic disease, chronic liver problems, and gall bladder diseases.

Taro is rich in mucilage (3–19%), and the content will depend on the extraction method [36]. In addition, mucilage has excellent viscosity, water-holding capacity, oil-holding capacity, and antimicrobial activity. Thus, these remarkable functional properties make taro mucilage a promising ingredient, with possible applications as a fat replacer [37], gel former [38], thickener, and emulsifier [39], Chemically, mucilage is a physiological product of plant metabolites and is composed of polysaccharide units of L-rhamnose, D-galactose, D-xylose, and L-arabinose. Mucilage also contains organic acids and a small number of proteins [39].

In contrast to other root crops, taro has been found to possess the highest levels of dietary fiber. For instance, raw and cooked taro corms contain approximately 13.5% and 3.21% fiber, respectively [18,27]. The chemical structure of non-starch polysaccharides in taro has been associated with immunomodulatory activities. Thus, Liu et al. [40] purified two new polysaccharides from taro corms (TPS-1 and TPS-2) and tested the immune activity of these polysaccharides. They concluded that TPS-1 and TPS-2 could be novel potential immunostimulants as both food and pharmaceutical supplements to improve immunity. Crude fiber is another nutritional benefit of taro with functional properties, including aiding in the delivery of micro-components and the digestion of glucose, slowing the cycle of reabsorption of unhealthy dietary components, such as cholesterol, reducing intestinal transit time, reducing LDL cholesterol in the blood, preventing constipation, raising food holding ability in the water, and decreasing blood glucose and insulin levels [41]. 

### 3.3. Protein 

Taro may not constitute a primary source of protein, with contents of 11% and 1.4% to 3.0% in dried and fresh taro, respectively; however, it is higher than the protein content in other root crops [21]. Taro (tubers) contains a distinctive protein polypeptide composition that has not been found in other root crops [42]. Notably, taro contains two significant types of proteins: mannose-binding lectin (MBL) and trypsin inhibitor [18,43]. However, deficiency in MBL has been linked to autoimmune syndromes, and the presence of trypsin inhibitor contributes to producing hypoallergenic proteins. Consequently, taro emerges as a promising alternative for individuals with food allergies [18]. According to a scientific study, the protein in taro is rich in all the essential amino acids, except histidine and lysine [44]. Varietal characteristics, environmental factors (such as temperature, moisture, and humidity), cultivation practices (such as fertilization and irrigation), and handling (such as storage) all affect protein content.

### 3.4. Vitamins and Minerals 

Many compositional studies of taro nutrition indicate that it contains many essential macronutrients and micronutrients. The taro corms and leaves contain significant amounts of vitamins C and B complexes, which are necessary for human nutrition [21]. In addition, beta carotene, iron, and folic acid can be found in taro leaves, which help to prevent anemia [45]. Though ascorbic acid and carotene are relatively weak sources of taro corms, their carotene quality is comparable to cabbage and twice as much as in potatoes. Furthermore, yellow-fleshed taro corm contains a higher amount of beta-carotene than the white-fleshed variants. Foods containing higher carotenoid concentrations effectively treat chronic diseases, including cancers, cardiovascular disease, and diabetes [21]. Table 3 presents the vitamin compositions in different taro parts from various studies.

Experiments on the compositions reported that taro is a significant source of potassium (3.23–5.30 g/kg fresh product). Other minerals present in the fresh product include magnesium (190–370 mg/kg), phosphorus (72.21–340 mg/100 g), sodium (0–3 mg/100 g), iron (8.66–10.8 mg/100 g), zinc (2.63 mg/100 g), copper (1.04 mg/100 g), and calcium (110–450 mg/kg) [50]. Zinc deficiency is one of the most common nutritional problems globally, and has an adverse effect on health, which is associated with stunting. However, as taro is an excellent non-animal source of zinc, its use should be encouraged to mitigate zinc deficiency [51]. Table 4 presents the mineral composition in different taro parts from different studies.

## 4. Antinutrients in Taro 

Antinutrients are naturally occurring substances that can prevent the body from absorbing or using some nutrients and are present in many plant-based foods. While plant-based foods offer health benefits, such as antioxidant properties, they can also pose challenges to nutritional availability and bioavailability. If ingested in excess or for an extended length of time, these compounds may result in nutrient shortages or other health problems. Oxalates, phytates, tannins, protease inhibitors, and polyphenols are a few examples of the antinutrients frequently found in fruits and vegetables.

Taro contains several antinutrients that can affect its nutritional value. Oxalates are found in taro leaves and corms and can form insoluble crystals that form kidney stones and reduce calcium absorption. Taro also contains trypsin inhibitors (TI), which can interfere with protein digestion and absorption. Additionally, taro contains phytates that can inhibit the absorption of minerals such as iron, zinc, and calcium. For example, taro is noxious when raw and considered toxic due to calcium oxalate crystals, which present a content of up to 2.05 to 4.21% in the dry matter [21,53], typically as raphides. Holloway et al. [54] reported that oxalate concentrations of 278–574 mg/100 g and 65 mg/100 g in fresh taro leaves and corms, respectively. Moreover, Huang and Tanudjaja [55] showed that taro corms contained 43–156 mg of oxalate/100 g of fresh mass. 

Consumers may experience itching and severe tissue irritation after eating taro-based foods due to their acrid elements. Acridity can even be caused by cooked leaves and petioles. Increased acridity may also result from environmental adversity (drought or nutritional stress), which can be experienced by the same cultivar while it grows. Presumably, itchiness arises when the calcium oxalate crystals are released and inflict minute punctures to the skin, when in contact with it. Bradbury and Holloway [56] suggested that the crystals interact with a particular chemical on the raphide surface before acridity is experienced. The acridity or irritation of taro corm poses a challenge to using taro flour as a value-added product. 

The acridity factors can be reduced by different common operations, such as peeling, grating, frying, soaking, fermentation, ethanol extraction, and cooking, especially with a pinch of baking soda and by steeping taro roots in cold water overnight [21]. The remotion of the thick skin layers may eliminate acridity, while a prolonged cooking time is needed [57]. Fermentation can result in a significant decline in the cocoyam flour (*Colocasia esculenta*) oxalate levels (58 to 65%). 

The calcium oxalate level often changed significantly after baking in wheat-taro composite cookies. For instance, a study reported that the baking process significantly raised the total oxalate concentration in taro [58]. In addition, some previous studies on taro also reported that baking could increase the concentration level of oxalates in food by evaporating water [59]. Boiling taro corm has been suggested to reduce oxalate content and rapidly remove taro corm irritation [58,60]. From these findings, it has been suggested that parboiled taro flour can be used as the starting material for food formulations. Changes in the oxalate structures were documented during precooked taro flour production [60].

The study conducted by Aboubakar et al. [60] revealed that boiling significantly reduced the oxalate content, likely due to leaching in the boiling solution and acid hydrolysis. Taro contains various antinutritional components, including trypsin inhibitors (TI), oxalic acid, and cyanide [21]. Microwave baking was found to be highly effective in inactivating trypsin inhibitors in taro, while flours made from dried chips, grated, or ground taro tubers showed no trace of TI activity. The stability of the TIs varied among different taro types, with lower temperatures showing more pronounced effects. 

Nevertheless, these antinutrients can be reduced through appropriate cooking methods, such as boiling, soaking, and fermentation. It is important to consider the antinutrient content in taros in the context of a balanced diet, despite its overall nutritional value.

## 5. Health Benefits

The consumption of roots and tubers as a source of carbohydrates has been proven to be advantageous for people with celiac disease or other allergic reactions due to their gluten-free nature. Taro has been found to contain several active compounds, such as resistant starch, mucilage, anthocyanins, hemagglutinin, non-starch polysaccharides, protein, tarin lectin, and others, which exhibit numerous beneficial properties, including antitumor, antimetastatic, antioxidant, and anti-inflammatory effects, as revealed by studies conducted by Li et al. [61]. Furthermore, taro is an excellent alternative for people who are allergic to milk or cereal products [62]. These findings highlight the potential health benefits of consuming taro and its traditional medicinal uses (Table 5), which call for further investigation. Overall, taro is a versatile and nutritious food with both nutritional and medicinal benefits, thereby making it a promising food for future research and development.

### 5.1. Antioxidant Activity

Antioxidants are naturally occurring substances in plants, such as phenolic acids and flavonoids, which help prevent the oxidation of biomolecules and reduce oxidative stress produced by free radicals. Free radicals are unstable molecules that can be produced during regular metabolism or in response to environmental factors, such as pollution and radiation. To prevent cell damage, antioxidants stabilize these molecules by donating electrons. This is crucial because free radicals have the potential to harm cells and contribute to chronic illnesses such as cancer, heart disease, and Alzheimer’s. When the balance of free radicals to antioxidants shifts in favor of free radicals, oxidative stress occurs, which can lead to cell damage and the emergence of chronic diseases. Antioxidant-rich diets may help lower the risk of chronic illnesses, such as cancer and heart disease. It is important to maintain a balanced and varied diet to ensure adequate intake of antioxidants and other necessary nutrients. 

Colocasia leaves, also known as taro leaves, are rich in bioactive chemicals that possess potent antioxidant properties, which make them effective against both biotic and abiotic stress, as evidenced by Nur-Hadirah et al. [76]. The total phenolic content (TPC) of Colocasia leaves has been extensively studied, and they have been found to contain approximately 250.23 mg gallic acid equivalent (GAE) per 100 g of fresh weight (FW) of TPC [64]. Microwave cooking has been shown to affect the TPC of Colocasia leaves, according to Singh et al. [77], microwave cooking with and without salt (5% *w*/*v*) resulted in a TPC of 242.6 mg GAE/100 g for Colocasia leaves, which was comparable to the TPC of other leafy vegetables. These authors also investigated the total flavonoid content (TFC) of 25 green vegetables and found that fresh Colocasia leaves had a TFC of 154.4 mg RE/100 g, which was higher than in chickpea leaves (148.9 mg RE/100 g). Gonçalves et al. [63] investigated the phenolic composition of three different Colocasia leaf varieties (white, red, and white-gained) under various irrigation settings (cold/hot water and drip irrigation) and found that all samples contained flavones, including mono-C-glycosides, di-C-glycosides, mono-C-(O-glycosyl) glycosides, derivatives of luteolin, apigenin, and chrysoeriol. Li et al. [71] identified ten chemicals in Colocasia leaves, including orientin, isoorientin, tryptophan, vitexin, isovitexin, luteolin-7-O-glucoside, luteolin-7-O-rutinoside, 1-O-feruloyl-D-glucoside, 1-O-caffeoyl-D-glucoside, and rosmarinic acid. In addition, boiling Colocasia leaves in salt water resulted in a significant increase (34.3%) in TFC concentration compared to leaves heated in water without salt, indicating an increase in free flavonols after boiling. Furthermore, Lloyd et al. [78] found that Colocasia leaves irrigated with higher amounts of NaCl (200 mM) contained more flavonoids than those irrigated with lower amounts.

To evaluate the antioxidative potential of Colocasia leaf varieties, such as giant white, red, and white, their DPPH, superoxide radicals, and nitric oxide scavenging activities were measured. The IC50 values for these varieties were in the range of 0.226–1.140 mg/mL, 0.076–0.145 mg/mL, and 0.521–1.485 mg/mL, respectively, which were significantly (*p* ≤ 0.05) lower than the values for standard ascorbic acid (7.1 mg/mL DPPH activity, 0.425 mg/mL superoxide radical activity, and 0.250 mg/mL nitric oxide activity) [63]. In a separate study, El-Mesallamy et al. [75] evaluated the antioxidant activity of Colocasia leaf extract by measuring its capacity to scavenge DPPH free radicals. They reported an IC50 value of 22 μg/mL, which was higher than standard ascorbic acid (11.4 μg/mL). 

### 5.2. Anticancer Activity

Cancer occurs when cells in the body grow and divide uncontrollably to form a mass of abnormal cells, called a tumor. Evidence suggests that free radicals may play a role in the development of some types of cancer. Free radicals are highly reactive molecules that can cause damage to cells, including damage to DNA, proteins, and cell membranes. When free radicals damage DNA, it can lead to mutations and other genetic changes that may contribute to the development of cancer. Taro contains a variety of bioactive compounds, including polyphenols, flavonoids, and carotenoids, which have been found to have potential anticancer properties. These bioactive compounds have been shown to have anticancer effects by inducing cell death and inhibiting the growth and proliferation of cancer cells. Some studies have found that polyphenols from taro may be effective against several types of cancer, including breast, colon, and liver.

In their study, Corrêa et al. [65] aimed to develop a liposomal nanocapsule formulation containing purified tarin, which is a lectin found in taro corms and has the potential to be an immunomodulatory and antitumor agent. The researchers were able to achieve high tarin entrapment rates (>80%) and minimal leakage (≈3%) during a 40-day storage period at 4 °C. Neither free nor encapsulated tarin showed any in vitro toxicity against healthy mouse bone marrow and L929 cells, although both were found to stimulate the production of fibroblast-like and large round-shaped cells. Encapsulated tarin demonstrated effectiveness in inhibiting the proliferation of human glioblastoma (U-87 MG) and breast adenocarcinoma (MDA-MB-231) cells, with IC50 values of 39.36 and 71.38 μg/mL, respectively. Notably, the efficacy of encapsulated tarin was found to be comparable to that of conventional chemotherapy drugs, such as cisplatin and temozolomide.

In 2015, Pereira and colleagues [8] conducted a study on tarin, which had been extracted from unrefined taro extract. The experiment found that tarin contained 2–3% carbohydrates and was formed of at least ten isoforms with a pI range from 5.5 to 9.5. They also discovered that tarin has a stable structure and maintains its functional properties even after exposure to various temperatures and pH levels. The researchers observed that tarin selectively binds to complex N-glycans and high-mannose, with a high affinity for several ligands that are present in insects, tumor cells, viruses, and hematopoietic progenitor cells. This study identified the structure and ligand-binding properties of a GNA-related lectin that has potential applications as a therapeutic molecule to promote immune response and proliferation.

Kundu et al. [66] have identified a promising therapeutic drug that targets metastatic breast cancer with low toxicity. The drug is derived from the edible root of the taro plant and has been shown to have demonstrable action in preclinical models of metastatic breast cancer. 

Recent research has shown that a water-soluble extract of taro (TE) significantly inhibits the lung-colonizing ability and spontaneous metastasis from mammary gland-implanted tumors in a murine model of highly metastatic breast cancer [66]. In addition, TE treatment resulted in moderate inhibition of several breast and prostate cancer cell lines, with observed morphological alterations and a complete cessation of cancerous cell migration. Furthermore, the administration of TE reduced the expression of cyclooxygenase 1 and 2 mRNA and lowered the production of prostaglandin E2 (PGE2). These observed effects are linked to three taro proteins: the 12 kDa storage protein, tarin, and taro lectin. Notably, these proteins possess a carbohydrate-binding domain and exhibit similar amino acid sequences [18]. This is a significant finding as it is the first report of taro-derived chemical(s) that effectively and precisely prevent tumor spread. This promising discovery could provide a new avenue for developing novel treatments for metastatic breast cancer with low toxicity.

### 5.3. Anti-Inflammatory Activity

One of the reported benefits of taro is its anti-inflammatory properties, which have been studied in various experiments. Methanolic and ethanolic extracts of *Colocasia esculenta* Linn. leaves have been explored for their potential anti-inflammatory effects on carrageenan-induced rat paw edema and lipopolysaccharide (LPS)-stimulated RAW264.7 cells. Taro has been found to contain several compounds with anti-inflammatory effects, including polyphenols, flavonoids, and anthocyanins, known for their antioxidant properties. Additionally, taro contains compounds such as saponins and alkaloids that have been found to have anti-inflammatory effects in various studies (e.g., [18]).

Agyare et al. [67] conducted a study investigating the anti-inflammatory effect of taro leaf extract on seven-day-old chickens. To evaluate the anti-inflammatory property of taro leaves, the researchers induced inflammation in the footpad of chicks using carrageenan. The extract was administered orally to the chickens at doses of 30, 100, and 300 mg/kg of body weight. The results showed that the doses of extract tested at 30 and 300 mg/kg of body weight were more effective in reducing inflammation than a dose of 100 mg/kg of body weight. The findings were statistically significant (*p* < 0.001) throughout the duration of the experiment. 

Another study by Baro et al. [68] examined the potential anti-inflammatory properties of Colocasia esculenta methanolic root extract (CEMRE) on rat paw edema. The results demonstrated that pretreatment with CEMRE at a dose of 400 mg/kg significantly reduced inflammation in the rat paw, induced by carrageenan injection, and in lipopolysaccharide (LPS) stimulated RAW264.7 cells, while this reduction was comparable to the standard drug Indomethacin.

In a previous study, researchers investigated the potential anti-inflammatory properties of the ethanolic extract of *Colocasia esculenta* Linn. leaves in Wistar rats with carrageenan-induced left hind paw edema [69]. The extract was administered orally at a dosage of 100 mg/kg and resulted in the inhibition of the edema. The findings demonstrated a significant anti-inflammatory effect by the extract compared to the standard and untreated control groups, with a significance level of *p* < 0.05.

Overall, the anti-inflammatory properties of taro (leaves and corm) make it a promising natural remedy for various inflammatory conditions, such as rheumatoid arthritis, inflammatory bowel disease, and other chronic inflammatory diseases. However, more research is needed to fully understand the mechanisms of action and potential therapeutic applications of taro for inflammatory conditions.

### 5.4. Antidiabetic Activity

Diabetes is a chronic disease, which affects how the body uses blood sugar (glucose). Diabetes has long been treated with insulin and hypoglycemic medications. However, in addition to being pricey, these drugs have several adverse effects. As a result, research has begun to concentrate on natural substances or plants with antidiabetic action rather than allopathic medications [79,80].

During diabetes, alterations in organ weights and metabolic processes have been observed. In particular, the kidneys have been reported to increase in weight due to excessive glucose utilization and subsequent enhancement in glycogen synthesis. Conversely, the liver has been observed to decrease in weight due to enhanced catabolic processes, which are attributed to the lack of insulin in liver cells. To investigate the potential ameliorating effects of incorporating cocoyam (*Colocasia esculenta* L.) into the diet, Eleazu et al. [70] conducted a study on diabetic rats induced with Streptozotocin (STZ).

To induce diabetes, rats were administered STZ, and those with fasting blood glucose levels exceeding 200 mg/dL after 7 days were considered diabetic. Then, the diabetic rats were divided into groups, with 1 group receiving a feed composed of 77% cocoyam flour. Prior to the study, the cocoyam-incorporated feed underwent phytochemical analysis, revealing an average composition of 2.65% flavonoids, 1.01% alkaloids, 0.70% saponins, and 1.06% tannins. These phytochemicals have been associated with hypoglycemic activity, making cocoyam a potentially valuable dietary component for managing diabetes. The administration of the cocoyam-incorporated feed to the diabetic rats resulted in a significant decrease of 58.75% in hyperglycemia (glucose: −ve to trace), compared to the diabetic control group and non-diabetic rats. Furthermore, the relative kidney weights of the diabetic control rats were significantly higher than the non-diabetic and diabetic rats treated with cocoyam (*p* < 0.05). These findings suggest that incorporating cocoyam into the diet may have a protective effect on kidney growth in diabetes. The presence of various phytochemicals with hypoglycemic properties in cocoyam flour provides a promising avenue for utilizing plant-based approaches, to mitigate the complications associated with diabetes.

A previous study by Li et al. [71] aimed to assess the impact of 95% ethanol extracts from *C. esculenta* leaves and its organic solvent soluble fractions on rat lens aldose reductase activity. The findings of this study suggest that two flavonoids obtained from *C. esculenta* leaves have potential therapeutic benefits in preventing and treating diabetic complications by preventing sorbitol accumulation in rat lenses through aldose reductase inhibition. Nevertheless, further clinical research is needed to substantiate their efficacy.

In a study conducted by Kumawat et al. [72], rats with alloxan-induced diabetes were given an ethanol extract made from the leaves of *C. esculenta* (EECE), and their blood glucose levels, and body weight were measured. These results indicate that in rats with alloxan-induced diabetes, EECE at a dose of 400 mg/kg displayed antihyperglycemic action.

In conclusion, the promising results obtained from the studies on the effects of taro (*Colocasia esculenta* L.) on diabetes management pave the way for future research in this area. Future investigations could delve deeper into the underlying mechanisms of taro’s hypoglycemic activity, to identify specific bioactive compounds responsible for its beneficial effects. Additionally, clinical trials involving human subjects could provide valuable insights into the potential application of taro as a dietary intervention for diabetes management.

### 5.5. Antimicrobial Activity

The possible antibacterial effects of taro have also been investigated. According to several studies, taro has antibacterial properties that make it effective against a range of pathogens, including bacteria, fungi, and viruses. In addition, the bioactive substances found in taro, including phenolic compounds, flavonoids, and alkaloids, have been demonstrated to have antimicrobial activities [74]. These substances are principally responsible for the taro’s antimicrobial qualities.

Wei et al. [73] studied the antibacterial activity of aqueous crude extracts in the taro leaf. This study showed that taro had potential against Gram-negative bacteria. The diameter (mm) of the inhibition zone was found to be 9, 9, 11, 12, 9, and 8 for *Citrobacter freundii*, *Vibrio alginolyticus*, *V. parahaemolyticus*, *V. harveyi*, *V. vulnificus*, and *V. cholerae*, respectively.

In their study, Chakraborty et al. [74] investigated the antimicrobial properties of taro tuber and leaves against nine different pathogens. They assessed the antimicrobial activity by measuring the zone of inhibition that formed after incubation. Methanol extracts from both the tuber and leaves showed a distinctive area of inhibition against all the tested pathogens. The researchers used different concentrations of the methanol extracts, ranging from 25 to 100 mg/mL. The highest zone of inhibition was observed at a concentration of 100 mg/mL for the tuber extract against *Klebsiella* sp. (3 cm), while the leaf extract showed the highest activity against *Proteus mirabilis* at a concentration of 100 mg/mL (1.5 cm). Notably, the tuber extract demonstrated more significant antibacterial activity than the leaf extract.

Elmosallamy et al. [75] studied the bioactive compounds, antibacterial, and antifungal activity of *C. esculenta*. The ethanol extract’s antibacterial efficacy against six clinical pathogens revealed a distinctive zone of inhibition. Comparing *Proteus vulgaris* (Gram-negative bacteria) to the conventional medicine Gentamicin, the maximum zone of inhibition was seen at a concentration of 100 mg/mL against *Proteus vulgaris* at 15 mm, while no effect was seen against *Bacillus subtitis*. Six different fungi were used to test the antifungal activity; the *Cryptococcus neoformans* inhibitory zone had the greatest value at 16 mm. Compared to the usual medication, ketoconazole at 100 mg/mL, the zone of inhibition was seen when using the 100 mg/mL concentration. According to the studies’ conclusions, the *Colocasia esculenta* leaf extract in a base of ethanol or methanol can potentially be an important component in treating bacterial and fungal infections. Therefore, further investigation should promote the use of herbal medicines in treating various illnesses by isolating and identifying the active ingredients responsible for their antibacterial and antifungal activities. Additionally, initiatives should be undertaken to create more specialized Colocasia esculenta extract-based medicines.

## 6. Food Products of Taro

Taro roots can be processed for consumption in various ways, including boiling, baking, roasting, frying, or incorporating them into other food preparations. Taro has a wide range of popular products, such as chips, dehydrated stable products, starch, flour, fufu, poi, achu, sapal, and even beer [81]. Table 6 presents the usage of different edible parts of taro in food products.

Among the different methods of taro preservation, producing flour and starches has been recognized as one of the most efficient approaches [21]. Taro is extensively utilized in the formulation of various food items, including taro bread or rolls, taro pancakes, and Kulolo (a fudge-like Hawaiian chocolate candy), where it serves as a primary component [81]. Continued advancements in technology have the potential to enhance the appeal of taro-based products to consumers. Extensive research has been conducted by various scientists on the utilization of taro as a nutritional component in canned foods and extruded products. This includes exploring its application in baby foods, taro flour, taro meal or grits, canned taro products, and extruded products such as rice, noodles, and macaroni, as well as its incorporation into fermented alcoholic beverages and as a substitute for gum in food products [21].

In different countries, various types of taro-based goods are produced. For example, in Taiwan, flakes and frozen cakes made from taro are popular, while in Ghana, taro flour is commonly utilized. China produces taro chunks, while America incorporates taro into infant formulas, and dried taro chips are a prevalent product in Fiji [21,81].

### 6.1. Food Ingredients

#### 6.1.1. Flour

Taro flour processing methods involve various unit operations, such as raw material selection, cleaning, peeling, cutting, blanching, drying, milling, packaging, and labeling. The different combinations of these unit operations can provide consumers with different products from taro flour. 

Various methods have been employed in the production of taro flour from taro cormels in different studies. In the study by Kaur et al. [82], taro cormels were washed, sliced, blanched (using distilled water at 90 °C for 2 min), dried at 50 °C in a hot cabinet drier for 4 h, and subsequently ground using a laboratory mixer. The resulting material was sieved through a no. 72 sieve (British Sieve Standards) to obtain flour.

Ammar et al. [102] followed a different procedure for taro flour preparation. They cleaned and rinsed the taro cormels, peeled them, sliced them into 2–3 cm thick pieces, and dried them at 45 °C in an air dehydrator for 24 h. Then, the dried slices were milled and passed through a no. 60 sieve (British sieve standards) to obtain flour.

In the study by Ikpeme-Emmanuel et al. [103], taro flour was prepared through a distinct process. Taro slices, obtained by peeling, slicing (1 mm thick), and washing, were soaked overnight in portable tap water (using a water volume twice that of the slices). After washing again, the slices were immersed in a 0.25% sodium bisulfate solution for 3 h. Then, the slices were divided into 2 batches: 1 batch was blanched by immersing in boiling water for 4 min, drained, and dried in an oven at 60 °C for 12 h. The dried slices from both batches were milled and sieved through a 60-mesh screen sieve (British standard) to achieve a sieve size of 0.250 mm.

Nip et al. [104] peeled, sliced, air-dried (60 rpm/20 h), and freeze-dried (60 rpm/24 h) the corms, followed by milling (Fitz mill) to 0.18 mm. While the production of taro flour might be slow because of the thick and stiff nature of the dried taro pieces, the pre-gelatinization process helps to change the characteristics of starch. Moreover, the variety and maturity level at the harvest affects the gelatinization temperature of the taro starch.

In the Pacific region, the processing of precooked taro flour typically involves boiling the tubers to achieve a soft texture, followed by subsequent drying and grinding. Uncooked taro flour demonstrates more uniform drying characteristics compared to precooked flour. The drying process of taro flour, predominantly obtained from gelatinized slices at elevated temperatures, significantly impacts its color and enhances its susceptibility to gelatinization [105]. Taro flour products present a viable substitution for rice in various countries worldwide, thereby contributing to the diversification of the food supply. Notably, taro flour exhibits distinctive properties, including small starch granules and a high mucilage (gum) content, which make it well-suited for the development of alternative bread and noodle formulations as substitutes for traditional wheat or corn starch-based products [82]. Industrially, taro flour finds application in the formulation of infant formulas, pastry fillings, sausage binders, and food emulsifiers [41,81]. By manipulating the temperature and moisture content of the dough, taro flour can be extruded into noodles [21]. Taro flour is commonly utilized in various food products worldwide, such as cakes in the Philippines and Colombia, bread in Brazil, and taro sticks in Indonesia [106]. Several researchers have explored the incorporation of taro flour into gluten-free bread formulations, aiming to assess its potential applicability [102,107].

#### 6.1.2. Starch

Taro is known to contain starch with a starch content ranging from 70% to 80% [108]. Taro starch can be utilized in its original form or serve as a raw material for various downstream processes and applications. Taro starch is gluten-free and has a smaller granule size than other root crops, which is associated with easy digestibility. Thus, taro has been used for infant formulation in Hawaii for children who are allergic to cereals and milk or milk products [41]. 

Currently, there are various enzymatic and conventional approaches being employed to extract starch from corms or cormels, which consequently impact both the yield and functional properties of the starch [98]. According to the referenced literature, the maximum yield achieved through conventional methods such as wet, centrifugal, and simple techniques ranged from 7 to 18.6% (on a wet basis), influenced by factors such as the botanical source, genetic factors, variety, harvest stage, granular size, and isolation method [109]. In a separate study by Sit et al. [110], an optimized enzymatic process utilizing cellulase and xylanase was implemented to extract and isolate starch from taro tubers and increased the yield by 14% to 18%, with a subsequent decrease in time and temperature of processing. 

The starch extracted from taro corms has a polygonal shape and presents fine granules (0.5–5 microns) [111], which produce a smooth gel. Additionally, it was also confirmed that fine granule starches enhance the binding capacity and reduce the breaking possibilities of snack products [112]. Taro starch contains about 50% less amylose but higher amylopectin than other cereals (1:7 ratio), which forms a clear and soft paste [41]. The gelatinization temperature depends on the variety and the maturity at harvest. It is lower as the age increases, ranging from 63 to 73 °C [22]. The taro starch has also been used for industrial applications, such as pasta, noddle, bakery, and biodegradable composite filling agents, and in the production of biodegradable plastics [81,113]. 

Some previous studies have reported the application of taro starch in different food products; tomato ketchup as a texture improver; yogurt as an improver of nutritional qualities, viscosity, and water holding capacity; edible films as an improver of appearance, physical and mechanical characteristics, impermeability to CO_2_ and extend shelf-life [89,90,97]. In addition, taro is also used in tablet formulations and disintegration, reducing the disintegration time in pills; microcapsules with excellent defense, together with probiotic strains; binder due to the small size of granules [114].

There are still many approaches currently being explored to unlock the full potential of taro roots. Nevertheless, the practical implementation of these technologies remains a significant obstacle in their processing. It is imperative for the industry to conduct investigations focused on standardizing the methodology for modifying taro starch, as well as developing complementary techniques to enhance its versatility and extend its shelf life [114].

### 6.2. Traditional Dishes

The population’s relationship to each food may differ depending on the country or region. For example, in Japan, traditionally, poor people used to consume taro as a substitute for rice. Taro is considered a popular and traditional food of the Coptic minority culture of Egypt. For Polynesians, it is a culture of high social status [3]. Dasheen, eddoes, elephant ears, woot, kalo, suni, sawa, malanga, inhame, tannia, and cocoyam, are some of the different names for which taro is known in the Caribbean, Hawaiian, Samoan, and West Africa [115]. Each one of these cultures has found a way to cook different parts of the taro plant. 

#### 6.2.1. Poi

Poi, a traditional food derived from taro, holds cultural significance and is widely consumed in Hawaii and the Pacific Islands [116]. The preparation of poi involves mashing cooked taro corms with water to form a starchy paste. The desired consistency is achieved by soaking taro roots in water and filtering them through cloth [116]. The natural fermentation of poi occurs when yeast and lactic acid bacteria present on the surface of the taro plant initiate the process, resulting in a tangy flavor [92]. Typically, this fermentation stage, without the use of starter cultures, takes approximately three days. Like the fermentation techniques utilized in making yogurt or sauerkraut, fresh poi undergoes a similar transformation. Throughout the whole process, the pH of poi steadily drops from 6.3 to 4.5 as acids are produced, with the lowest pH typically occurring around the 5th day [18]. Despite its extended fermentation period, poi remains a popular staple food. Lactic acid bacteria, including Lactococcus lactis, Lactobacillus plantarum, Leuconostoc lactis, Tetragenococcus halophilus, and Weissella confusa, play a crucial role in this process, enhancing the taste, aroma, and shelf life of the product [117]. Brown and Valiere [116] speculated on the probiotic potential of poi, as it primarily consists of lactic acid-producing bacteria, notably Lactococcus lactis (95%) and Lactobacilli (5%), with significantly higher levels per gram compared to yogurt. Additionally, studies conducted in Asia have demonstrated that infants fed with poi, a taro-based baby food, experience fewer health issues, such as diarrhea, pneumonia, enteritis, and beriberi, compared to those fed with rice and bread [21]. Moreover, Darkwa and Darkwa [81] highlighted the nutritional value of poi, emphasizing its hypoallergenic nature, an abundance of calcium, potassium, phosphorus, magnesium, B vitamins, vitamins A and C, high fiber content, and its role as a slow-release energy source.

The outstanding digestibility of poi appears linked to its efficient breakdown process. Previous research suggests that the rapid fermentation of poi contributes to enhanced mineral absorbability, particularly for phosphorus and calcium, and facilitates straightforward digestion [18,118]. Additionally, a separate study involving human participants found no evidence of undigested fiber in fecal samples following high consumption of poi [18,116].

#### 6.2.2. Sapal

Sapal is a type of traditional fermented taro dish, almost jelly-like, which is popular on the northern coast and some islands of Papua New Guinea. It is made with cooked taro corm and coconut milk at a ratio of 5:1 and allowed to ferment at room temperature. Taro fermentation into sapal was produced primarily by lactic acid bacteria, such as *Leuconostoc mesenteroides* or *Leuc. Paramesenteroids* exceeded final product concentrations of 1.6 to 10 CFU/mL [119]. Sapal is a traditional beverage that is occasionally produced in large quantities for social gatherings and the exchange of presents with neighbors. It is also stated as an alcoholic drink produced by colocasia fermentation that utilizes steamed fresh colocasia bulbs [120].

In Cameroon, taro produces a much sought-after snack, known as Achu, which is regarded as a digestible diet. However, the preparation of Achu is constrained owing to the difficulty of the processing, particularly its long processing time, owing to the pounding of the corms and the lack of mechanization. Achu is traditionally prepared by cooking fresh corms in boiling water until they are soft, peeling them, and smashing them in a mortar until they form a smooth and uniform paste [84].

### 6.3. Baked Products

Bakery products have been widely consumed and have become a significant component of the global food market [121]. 

The use of taro in bread-making has been the subject of many investigations. Taro tuber and roots are free from gluten, making them suitable for individuals with celiac disease or other allergic responses [122]. These favorable attributes of taro create opportunities for its utilization in gluten-free formulations. The demand for alternatives to wheat flour is challenging since wheat flour possesses distinctive viscoelastic properties primarily attributed to its major protein component, gluten. 

A previous study by Ammar et al. [102] investigated the influence of taro flour on bread production (Egyptian bread). They reported that up to 10% of the substitution of wheat flour had the same textural and sensory properties compared to regular bread. 

In another study by Abera et al. [118], the addition of taro flour to the bread formulation as a substitute for wheat flour was examined. The authors concluded that the acceptability of taro–wheat bread decreased as the blending ratio of taro flour increased, mainly due to the presence of a salty taste and unusual flavor in the blended bread. 

Furthermore, Arici et al. [87] conducted research on the utilization of taro flour as a substitute for wheat flour in the production of gluten-free bread. Their study demonstrates that taro flour exhibits considerable potential as an ingredient in bread formulations, not only for its ability to serve as a suitable replacement but also for its capacity to enhance the nutritional profile of the resulting bread.

Saklani, et al. [88] developed a cake enriched with taro in different mixtures with wheat (10, 20, 30, and 40%). These authors conclude that the inclusion of composite taro flour at a 40% replacement level resulted in cakes with physical properties comparable to those made with only wheat flour. Notably, the taro–wheat composite flour demonstrated the lowest setback and processing stability, indicating reduced staling or aging of the cake dough when taro flour was used as a replacement ingredient.

The study conducted by Alflen et al. [96] investigated the impact of partially replacing wheat flour with taro flour on the physical, nutritional, and sensory properties of cookies. The sensory analysis revealed no significant differences in the sensory attributes between the control cookies and those incorporating taro flour. The study demonstrated the feasibility of substituting up to 30% of the wheat flour with taro flour in the production of cookies.

These findings highlight the potential of taro as a valuable ingredient in the development of innovative and healthier bakery products. Further research and exploration of taro’s functional properties can contribute to the diversification and expansion of gluten-free options in the bakery industry.

### 6.4. Extruded Snacks 

Extrusion technology is based on forcing the food materials to form the desired shape through a barrel at high temperatures and pressures that pass through a die at the end of the barrel. According to [123], the food extruder is a high-temperature, short-time synthesizer that converts various raw food materials into completely modified products.

Only a limited number of studies have been conducted on taro flour and its usage in extruded products. However, in an experiment by Maga et al. [124], they processed taro flour and used it to produce snacks. Those products showed a significant expansion but had an unpleasant odor and taste. Rodriguez-Miranda et al. (2010) analyzed extruded snacks made from a mixture of taro and nixtamalized or non-nixtamalized maize flour and reported that the puffed snacks had a good score in consumer acceptability. 

Based on the physical properties, extrudates from taro flour produced desirable values concerning their expansion, density, color, and breaking strength compared to sweet potato and potato extrudates. Furthermore, taro extrudate scored the highest overall acceptability from the organoleptic evaluation. Therefore, taro extrudate has the potential for acceptance as a snack. Furthermore, it can increase its protein level by incorporating other high-protein materials. such as legume flour, by adding prawn or fish powder in the formulation, or by dusting on the final product to cater to specific consumers. Additionally, it is possible to increase the fiber content by adding dietary fiber from vegetable sources. Therefore, this could be an effective way to use root tubers to produce extruded snacks [125]. Pensamiento-Niño et al. [126] optimized the process to develop an extruded snack based on taro flour (*Colocasia esculenta* L.), enriched with mango pulp (*Mangifera indica* L.), and prepared using a single screw extruder to evaluate the effect of extrusion temperature, feed moisture content, and the proportion of mango pulp in taro flour on some process parameters, physical, functional properties, and b-carotene content of the extruded snack.

#### Taro Noodles

Noodles are a staple food eaten in Asian countries. Several different kinds of noodles exist due to variations in the composition, preparation method, and presentation according to regional preference [21]. Noodles are a food product that is made by stretching, extruding, or rolled-flattening and cutting from unleavened dough. Noodles can be refrigerated in their fresh form for short-term storage or stored dry for a long time, mainly for marketing. They are commercialized worldwide in various forms and shapes, including the most commonly long and thin stripes, while other shapes may include waves, helices, tubes, strings, and shells.

In a recent study conducted by Afifah et al. [85], it was demonstrated that substituting taro flour and leaf puree in dry noodles had an impact on various nutritional components. The findings revealed a decrease in protein content and organoleptic quality. However, the selected noodle formula, which included 40% taro flour and 100% taro leaf puree substitution, met the quality requirements and showed an effect on glycemic load. This suggests that it could be a viable alternative for individuals with diabetes mellitus, considering its antioxidant activity and starch digestibility, while having no significant impact on the glycemic index.

It was shown that noodles provide alternative uses of tropical roots and tubers, such as taro. Furthermore, the partial substitution of wheat with taro flour provides different noodles and can also help lower the risk of different allergic reactions and increase the utilization of taro corms. 

### 6.5. Dairy Products

#### 6.5.1. Ice Cream

Identity standards in the United States require ice cream to contain a minimum of 10% milk fat content and 20% total milk solids [127]. However, Taiwanese consumers have classified traditional taro ice products as unique based on the flavor, smooth texture, and particular color they appreciate. As a result, taro ice products are now the object of industrial production worldwide, especially in the Pacific region [128].

Some studies have reported on the usage of cooked taro paste to substitute milk and other dairy ingredients in the formulation of ice cream [93,94]. Taro ice cream mainly consists of taro flour, hydrogenated vegetable fat, and sugar. Taro ice cream (8% fat and 1% protein) has similar nutritional benefits to frozen sorbet (1% fat and 0% protein), yet with taste and sensory attributes comparable to high-fat (18% fat and 4% protein) premium ice cream. However, some minor ingredients had to be used in this frozen taro dessert to prevent large ice crystals from forming. Furthermore, due to their high starch content, the patented technology suggested a longer time for taro ice cream to soften before serving. 

The processing of low-fat ice cream using taro corms, either fresh or boiled by substitution on ice cream mix until 30%, did not affect the final product characterized by the same properties as the full-fat product and had an increase in the antioxidant compound. Therefore, the authors recommended that the examined low-fat ice cream that had 30% boiled taro corms added was of higher quality after reducing the fat content and stabilizer/emulsifier complex by half [93].

#### 6.5.2. Taro Yogurt

Taro yogurt was the focus of some research in the United States in 1990 and was the most fascinating and innovative experiment of taro-based products. Taro yogurt has been renamed “taro gurt” in this respect, although this is still under discussion. The development of taro yogurt began with the discovery of Lactobacillus sp. (*Lactococcus lactis, Lactobacillus plantarum, Leuconostic lactis, Tetragencoccus halophilus, and Weissela confusa*) in natural poi in Hawaii [92]. They all form white pinpoint colonies, Gram-positive, catalase-negative, and facultative anaerobe. Such natural LAB (lactic acid bacteria) have been tested in yogurt production compared to the existing commercial fermentation of yogurt. The first time they tried to use commercially available LAB to make regular yogurt or sour milk, such LAB could not expand in the taro paste medium. The pH range was 4.29 to 3.03 in taro yogurt after 18 to 42 h of incubation [90]. Examination of the fermented medium showed that acetic acid and succinic acid had simultaneously accumulated alongside lactic acid. Conversely, when LAB isolated from poi were used as a starter, they grew much more vigorously. In particular, *Weissela confusa* produced the best growth and resistance in taro paste. However, most of these organisms did not resist the acidic pH, and it was difficult to maintain growth and the bacteria count after 3 days [129].

### 6.6. Other Food Products

Ikpeme Emmanuel et al. [100] developed an affordable and nutrient-dense weaning food using composite flours of taro and soybean. To achieve a protein content of 20%, comparable to other weaning foods, taro flour was extruded with whey protein concentrate (WPC), whey protein isolate (WPI), or lactalbumin (LAC). The study observed that taro extrudates without protein exhibited higher water absorption and solubility compared to products containing whey proteins or LAC. An infantile flour was prepared by combining taro (*Colocasia esculenta* (L.) Schott) and maize flours, which contain essential fatty acids (such as linoleic acid and oleic acid) and minerals (such as calcium, magnesium, iron, copper, zinc, phosphorous, potassium, chloride, sodium, and manganese) in varying concentrations. The weaning mush prepared from these mixed flours exhibited a high energy density of 119 kcal/100 mL [99]. Temesgen et al. [101] designed a pregelatinized taro flour and developed a weaning food, these authors observed that the weaning foods prepared from 25% taro flour provided better nutritional and functional compositions to meet the nutrient density of infant foods.

In the context of energy drinks, the utilization of taro as a carbohydrate supplement has been previously documented [97]. Initially, taro grit and sorghum grist were meticulously prepared, followed by comprehensive chemical analyses conducted to ascertain their respective nutritional profiles. Subsequently, various combinations of grit and malt grist were employed to create samples of energy drinks, with the objective of identifying the formulation that could closely replicate the sensory attributes of the established commercial standard. The standard commercial drink was composed of cocoa and malt. Remarkably, the total carbohydrate content of taro grit (77.93%) exhibited similarities to the carbohydrate content of cocoa (63–79%), thus, positioning taro as a promising source of carbohydrate supplementation in energy food beverages. Furthermore, the fat content (ether extract) of taro grit (0.63%) demonstrated comparability to the fat content found in cocoa (1.8–2.9%), indicating that taro is not only suitable as a carbohydrate supplement but also possesses a reduced susceptibility to rancidity compared to coco. In the sensory evaluation phase, participants evaluated the products based on their aroma and flavor, attributing similarity scores of 99.98% and 84.68%, respectively, to the commercial product. The taro-based drink exhibited a brown appearance, a pleasant odor, and a crisp texture, which aligned with the universally accepted standards for energy food beverages. These findings suggest that taro holds substantial potential for diversifying and enhancing the nutritional value of food products, thereby contributing to the amelioration of food insecurity and malnutrition.

Sit et al. [98] conducted a study to elucidate the physicochemical and functional characteristics of taro starch and to investigate its impact when incorporated into tomato ketchup alongside maize starch, focusing on the sensory qualities of the resulting product. The amylose content of taro starch was determined to be 18.14% on a dry basis, with a starch purity of 95.87%. Notably, the physicochemical properties of taro starch closely resembled those of maize starch. One vital consideration in starch selection is solubility and swelling, and taro starch exhibited higher values in these parameters. This makes taro starch suitable for applications requiring high-temperature heating, as it possesses a higher pasting temperature. Moreover, the decreased final and setback viscosities observed with taro starch indicate that it can function as an effective thickening agent in products requiring prolonged heating and stirring. Comparative analysis revealed that tomato ketchup containing 1% taro and maize starch had a smoother texture compared to the control sample or ketchup made with 2% taro and maize starch. Furthermore, the mouthfeel ratings of ketchup containing 1% and 2% taro starch, as well as 2% taro starch alone, were significantly higher compared to the control sample or tomato ketchup made with 2% maize starch. Panelists expressed that the ketchup made with 2% maize starch had a firmer and less appealing texture. Therefore, incorporating taro starch in tomato ketchup can enhance mouthfeel and texture without compromising its acceptability. The sensory evaluation demonstrated that the inclusion of taro starch significantly improved texture properties, such as firmness and consistency, without affecting the color of the ketchup. These improvements were comparable to those achieved with maize starch, indicating that taro starch holds promise for use in both food and non-food applications.

## 7. Conclusions

In conclusion, taro has gained recognition as a low-cost dietary energy source and staple food with immense nutritional, medicinal, and therapeutic potential. While it has been found to have antinutritional qualities, various processing methods can effectively reduce these elements, making taro more suitable for consumption. Taro has the potential to be a valuable food crop that can reduce food insecurity, especially in developing countries. However, research on taro storage and quality over time is limited, and the availability of suitable equipment for processing it on an industrial scale needs to be addressed. Additionally, more efforts are needed to increase the global reach of taro-based products, conduct research into potential applications, improve processing environments, and reduce post-harvest losses. Further study and funding are required to fully unlock the potential of taro in improving food security and economic growth, particularly in developing nations.

## Figures and Tables

**Table 1:** World taro production in recent years by region.

Year	Area Harvested (Ha)				Total Yield (Tons)			
	Africa	Asia	Oceania	Americas	Africa	Asia	Oceania	Americas
2000	1,250,204	128,872	44,361	7622	8,233,653.65	1,930,699.73	318,752.90	78,815.08
2005	1,383,172	127,935	55,659	8915	9,849,310.49	1,914,001.54	413,899.85	82,994.87
2010	1,194,840	132,284	52,462	4883	7,754,061.42	2,079,541.37	402,544.38	55,662.43
2015	1,575,862	147,251	48,310	7836	8,632,488.23	2,366,643.69	416,184.77	133,346.23
2021	1,590,820	148,515	48,048	6320	9,525,695.56	2,395,189.79	410,496.50	64,866.65

Source: FAOSTAT 2023 [14].

**Table 2:** Proximate compositions of different taro parts.

Compositions	References					
	Corm ^£^	Leaves	Corm	Corm	Corm	Corm
	[23]	[24]	[25]	[20]	[26]	[27]
Moisture (%)	6.21	88.5	63–85	72.4	71.7	70.90
Protein (%)	8.07	2.7	1.4–3.0	1.10	2.57	6.43
Fat (%)	0.45	n.d.	0.16–0.36	0.20	0.15	0.47
Fiber (%)	3.10	2.8 ^‡^	0.60–1.18 ^‡^	3.60	0.61 ^¥^	2.63
Ash (%)	2.78	2.5	0.60–1.3	1.0	1.37	4.82
Carbohydrate (%)	85.60	3.1	13–29	19.20	22.3	n.d.
Total energy (kcal/100 g)	n.d.	n.d.	n.d.	372.60	103	372.55

¥: soluble; ‡: crude; £: corm flour; n.d.: not determined.

**Table 3:** Vitamins compositions in different taro parts.

Compositions (mg/100 g)	References			
	Leaves	Leaves	Corm	Cormels
	[46]	[47]	[48]	[49] ^†^
Thiamin	0.209	0.08	0.02	0.21
Riboflavin	0.456	0.07	0.03	0.04
Niacin	1.51	0.80	0.78	0.58
Pantothenic acid	0.084	0.27	n.d.	n.d.
pyridoxine	0.146	0.29	n.d.	n.d.
Biotin	n.d.	12.10	n.d.	n.d.
Folate	0.126	0.159	0.022	n.d.
Vitamin A	0.241	n.d.	0.006	8.92
Vitamin C	52	40.71	14.30	10.29
Vitamin E	2.02	0.07	3	1.89

†: dry weight basis; n.d.: not determined.

**Table 4:** Mineral compositions in different taro parts.

Compositions (mg/100 g)	References					
	Leaves	Leaves	Corm	Corm	Corm	Edible Part
	[46]	[47]	[27] ^†^	[19]	[52]	[45] ^†^
Ca	112	216	45.23	86.70	782.15	137
Fe	2.35	3.41	5.86	1.16	218.50	0.20
Mg	47	59.44	7.24	100	543.90	23.70
P	63	57.88	7.77	1.39	n.d.	16.70
Na	133	12.08	13.81	n.d.	25.6	46.40
K	675	404	n.d.	224	372.40	141
Mn	n.d.	1.30	3.61	1.12	221.30	2.2
Zn	0.43	0.82	43.08	5.14	392.23	4.80
Cu	0.281	0.29	0.43	0.67	231.70	0.40
Se	0.0009	0.0043	n.d.	n.d.	n.d.	n.d.

†: dry weight basis; n.d.: not determined.

**Table 5:** Phytochemical properties and health benefits of taro.

Properties	Used Part	Bioactive Compounds	Results	Ref.
Antioxidant	Leaf	Mono-C-glycosides, di-C-glycosides, mono-C-(O-glycosyl) glycosides, derivatives of luteolin, apigenin, and chrysoeriol.	The IC50 values for three different varieties were in the range of 0.226–1.140 mg/mL, 0.076–0.145 mg/mL, and 0.521–1.485 mg/mL.	[63]
	Leaf	Phenolic compounds	The extract contained approximately 250.23 mg gallic acid equivalent (GAE) per 100 g of fresh weight.	[64]
Anticancer	Corms	Tarin	Encapsulated tarin demonstrated effectiveness in inhibiting the proliferation of human glioblastoma (U-87 MG) and breast adenocarcinoma (MDA-MB-231) cells, with IC50 values of 39.36 and 71.38 μg/mL, respectively.	[65]
	Corms	Lectin	The structure and ligand-binding properties of a GNA-related lectin that has potential applications as a therapeutic molecule to promote immune response and proliferation.	[8]
	Corms	12-kDa storage protein, tarin, and lectin	TE treatment resulted in moderate inhibition of several breast and prostate cancer cell lines, with observed morphological alterations and a complete cessation of cancerous cell migration. Furthermore, the administration of TE reduced the expression of cyclooxygenase 1 and 2 mRNA and lowered the production of prostaglandin E2 (PGE2).	[66]
Anti-inflammatory	Leaf	Steroids	The extracts possessed significant inhibitory effects on preformed mediators, such as histamine and serotonin, which are involved in the initial phase of the acute inflammatory process.	[67]
	Root	Sinapic acid	A dose of 400 mg/kg significantly reduced inflammation in the rat paw and lipopolysaccharide (LPS) stimulated RAW264.7 cells	[68]
	Leaf	Steroids	The extract was administered orally at a dosage of 100 mg/kg, resulting in the inhibition of edema.	[69]
Antidiabetic	Tuber flour	Flavonoids, alkaloids, saponins, and tannins	Significant decrease of 58.75% in hyperglycemia (glucose: −ve to trace), compared to the diabetic control group and non-diabetic rats. The relative kidney weights of the diabetic control rats were significantly higher than the non-diabetic and diabetic rats treated with cocoyam.	[70]
	Leaf	Flavonoids	Prevented and treated diabetic complications by preventing sorbitol accumulation in rat lenses through aldose reductase inhibition.	[71]
	Leaf	Flavonoids and tannins	Rats with alloxan-induced diabetes, extraction at a dose of 400 mg/kg displayed antihyperglycemic action.	[72]
Antimicrobial	Leaf	Folic acid alkaloids, glycosides, phenolics, and resins	The diameter (mm) of the inhibition zone was found as 9, 9, 11, 12, 9, and 8 for *Citrobacter freundii*, *Vibrio alginolyticus*, *V. parahaemolyticus*, *V. harveyi*, *V. vulnificus*, and *V. cholerae*, respectively.	[73]
	Tuber and leaf	Flavonoids, carbohydrates, tannins, and terpenoids	Methanol extracts from both the tuber and leaves showed a distinctive area of inhibition against all the pathogens tested.	[74]
	Leaf	-----	The ethanol extract’s antibacterial efficacy against six clinical pathogens revealed a distinctive zone of inhibition. Comparing Proteus vulgaris (Gram-negative bacteria) to the conventional medicine Gentamicin, the maximum zone of inhibition was seen at a concentration of 100 mg/mL against *Proteus vulgaris* at 15 mm.	[75]

**Table 6:** Recent research on food products produced by taro.

Food Product	Used Part	References
Flour/starch	Root/cormels	[82,83,84]
Noodle	Root flour	[21,85,86]
Cake	Root flour	[87,88]
Edible film	Underground stems/corms	[89]
Yogurt	Corms	[90,91,92]
Ice cream	Root flour	[93,94]
Bread	Root flour	[87,95]
Cookie	Root flour	[96]
Beverage	Cocoyam	[97]
Ketchup	Root starch	[98]
Weaning food	Taro Flour	[99,100,101]

## Data Availability

Not applicable.
