# Correction: Ferdaus et al. Taro Roots: An Underexploited Root Crop. *Nutrients* 2023, *15*, 3337

**DOI:** 10.3390/nu16131983

**Published:** 2024-06-21

**Authors:** Md. Jannatul Ferdaus, Ezzine Chukwu-Munsen, Aline Foguel, Roberta Claro da Silva

**Affiliations:** 1 Family and Consumer Sciences, College of Agriculture and Environmental Sciences, North Carolina A&T State University, Greensboro, NC 27411, USA; 2 Department of Biochemical-Pharmaceutical Technology, Faculty of Pharmaceutical Sciences, University of Sao Paulo, Sao Paulo 05508-000, SP, Brazil

## Text Correction and Missing Citation

The Editorial Office was made aware that the original publication [1] contained unattributed overlap with a doctoral thesis. The Editorial Office then completed an investigation in line with our policy (https://www.mdpi.com/ethics#_bookmark29, accessed on 5 June 2024). As a result of the investigation and discussions with the authors, the Editorial Board decided to issue a correction containing the changes described below.

## Text Correction

A correction has been made to the first sentence in the third paragraph of Section 3.2, which now reads as follows:

In contrast to other root crops, taro has been found to possess the highest levels of dietary fiber. For instance, raw and cooked taro corms contain approximately 13.5% and 3.21% fiber, respectively [18,27].

A correction has been made to Section 3.3 from the second to the fourth sentences, which now read as follows:

Taro (tubers) contains a distinctive protein polypeptide composition that has not been found in other root crops [42]. Notably, taro contains two significant types of proteins: mannose-binding lectin (MBL) and trypsin inhibitor [18,43]. However, deficiency in MBL has been linked to autoimmune syndromes, and the presence of trypsin inhibitor contributes to producing hypoallergenic proteins. Consequently, taro emerges as a promising alternative for individuals with food allergies [18].

A correction has been made to the fourth sentence in the fifth paragraph of Section 5.2, which now reads as follows:

These observed effects are linked to three taro proteins: the 12 kDa storage protein, tarin, and taro lectin. Notably, these proteins possess a carbohydrate-binding domain and exhibit similar amino acid sequences [18].

Ref. [18] has been inserted at the end of Section 5.3, paragraph 1, which now reads as follows:

Additionally, taro contains compounds such as saponins and alkaloids that have been found to have anti-inflammatory effects in various studies (e.g., [18]).

A correction has been made to the sixth and seventh sentences in the first paragraph of Section 6.2.1, which now read as follows:

Like the fermentation techniques utilized in making yogurt or sauerkraut, fresh poi undergoes a similar transformation. Throughout the whole process, the pH of poi steadily drops from 6.3 to 4.5 as acids are produced, with the lowest pH typically occurring around the 5th day [18].

A correction has been made to Section 6.2.1, paragraph 2, which now reads as follows:

The outstanding digestibility of poi appears linked to its efficient breakdown process. Previous research suggests that the rapid fermentation of poi contributes to enhanced mineral absorbability, particularly for phosphorus and calcium, and facilitates straightforward digestion [18,118]. Additionally, a separate study involving human participants found no evidence of undigested fiber in fecal samples following high consumption of poi [18,116].

## Update to References

Ref. [18] has been updated.

The updated reference appears below:

18. Saxby, S.M. The Potential of Taro (Colocasia Esculenta) as a Dietary Prebiotic Source for the Prevention of Colorectal Cancer. Ph.D. Thesis, University of Hawai’i at Mānoa, Honolulu, HI, USA, 2020.

The authors state that the scientific conclusions are unaffected. These corrections were approved by the Academic Editor. The original publication has also been updated.
